# Analysis of Lutein Content in Microencapsulated Marigold Flower Extract (*Tagetes erecta* L.) Using UHPLC-Q-Orbitrap-HRMS and Its Cytotoxicity in ARPE-19 Cells

**DOI:** 10.3390/molecules28166025

**Published:** 2023-08-12

**Authors:** Pornson Suwanklang, Thavaree Thilavech, Waree Limwikrant, Worawan Kitphati, Wasu Supharattanasitthi, Pattamapan Lomarat

**Affiliations:** 1 Department of Food Chemistry, Faculty of Pharmacy, Mahidol University, Bangkok 10400, Thailand; pornson.sua@student.mahidol.ac.th (P.S.); thavaree.thi@mahidol.ac.th (T.T.); 2 Department of Manufacturing Pharmacy, Faculty of Pharmacy, Mahidol University, Bangkok 10400, Thailand; waree.lim@mahidol.ac.th; 3 Department of Physiology, Faculty of Pharmacy, Mahidol University, Bangkok 10400, Thailand; worawan.kit@mahidol.ac.th (W.K.); wasu.sur@mahidol.ac.th (W.S.); 4 Centre of Biopharmaceutical Science for Healthy Ageing, Faculty of Pharmacy, Mahidol University, Bangkok 10400, Thailand; 5 Mahidol-Liverpool Joint Unit for Ageing Research, Faculty of Pharmacy, Mahidol University, Bangkok 10400, Thailand

**Keywords:** lutein, marigold extract, UHPLC-Q-Orbitrap-HRMS, cytotoxicity, ARPE-19

## Abstract

Organic solvents are commonly used to extract lutein. However, they are toxic and are not environmental-friendly. There are only a few reports on the quantification of lutein. Therefore, this study aimed to determine a suitable extraction method by which to obtain lutein from marigold flower (*Tagetes erecta* L.), using coconut oil to evaluate the cytotoxicity of extract in ARPE-19 cells, to optimize the encapsulation process for the development of microencapsulated marigold flower extract, and to develop the method for analysis of lutein by using UHPLC-Q-Orbitrap-HRMS. Coconut oil was used for the extraction of marigold flowers with two different extraction methods: ultrasonication and microwave-assisted extraction. The UHPLC-Q-Orbitrap-HRMS condition for the analysis of lutein was successfully developed and validated. Marigold flower extract obtained using the microwave method had the highest lutein content of 27.22 ± 1.17 mg/g. A cytotoxicity study revealed that 16 µM of lutein from marigold extract was non-toxic to ARPE-19 cells. For the development of microencapsulated marigold extract, the ratio of oil to wall at 1:5 had the highest encapsulation efficiency and the highest lutein content. Extraction of lutein using coconut oil and the microwave method was the suitable method. The microencapsulated marigold extract can be applied for the development of functional ingredients.

## 1. Introduction

Marigolds (*Tagetes erecta* L.) are plants with yellow flowers, belonging to the Asteraceae family. They are an important source of lutein. It was found that marigold flowers contain lutein content at around 70% of the carotenoid content [1,2]. Lutein is an excellent antioxidant that is used in the food, cosmetic, and pharmaceutical industries [1]. Marigold petals consisting of lutein and zeaxanthin have been used to enhance egg yolk colors; the higher yolk color score correlates with higher xanthophylls content [3]. Lutein is also associated with eye health because lutein is prominently present in the macular region, which is an important component of the eye. In addition, lutein is found everywhere in the eyes, especially the peripheral retina, retinal pigment epithelium, choroid, and ciliary body [1,4,5,6]. It acts as a pigment in UV filters and absorbs high-energy blue light. Nowadays, we have stepped into the digital era. The statistics on the use of communication tools, namely, smartphones, computers, and laptops, have been increasing progressively. The prolonged exposure to blue light from these devices can be harmful to the eyes [7] because blue light can damage the cornea, lens, crystal, and retina by penetrating through the lens to the retina [8]. The damage of retinal pigment epithelium (RPE) cells causes the degeneration of photoreceptors, which leads to visual loss; therefore, it is important to avoid and prevent those damages. Lutein consumption of 6 mg/day is associated with a reduced risk of age-related macular degeneration, and the intake of lutein 10 mg and 2 mg of zeaxanthin was found to be the appropriate ratio [5,8]. Therefore, supplementing lutein with food may be a good way to reduce the risk of visual impairment [9]. Currently, the heath claims of lutein on eye health still show inconsistency. However, it is undeniable that health products (functional foods and dietary supplements) containing lutein have recently been highly demanded by consumers, according to current, wide-ranging health and wellness trend reports. A variety of formulations or dosage forms of lutein used in the studies to prove its health benefits could lead to inconsistent data with respect to its health claims because different formulations or dosage forms can affect the stability of lutein and consequently influence the bioavailability and its efficacy. In addition to the need for more clinical trials to create stronger scientific substantiation for lutein health claims, studies on the improvement of lutein stability are also important in the development of health products such as functional foods and dietary supplement containing lutein.

Lutein (PubChem CID: 5281243; CAS number: 127-40-2; Molecular Formula: C_40_H_56_O_2_) is a lipophilic compound which can be extracted in non-polar solvents and presented in a limited amount in polar solvents [10]. Lutein is normally extracted from marigold flowers via different kinds of organic solvents such as hexane, acetone, and ethanol, but the most popular and effective solvents are toxic. The organic solvent has to be removed completely via evaporation to ensure safety in food applications [11]. Therefore, an alternative extraction method—especially green extraction, which is environmental-friendly—needs to be discovered. Extraction using edible oil as a solvent is another interesting method and has begun to be applied in some studies. Coconut oil is one of the most popular edible oils used for extraction. Certain health benefits have been claimed, such as antioxidant, anti-inflammatory, and lipid-lowering effects [12]. Coconut oil is the major source of medium-chain triacylglycerols, which is rapidly absorbed and utilized when consumed as compared to long-chain triacylglycerol. Therefore, it is burnt for energy rather than stored in the body. In addition, coconut oil is very stable against oxidation and does not easily form peroxide because of the high level of saturation compared to other edible oils [13,14]. Interestingly, lutein has some limitations due to its susceptibility to oxygen, temperature, and light; lutein may deteriorate during processing and storage. Similarly, most of the edible oils are often chemically unstable and susceptible to deterioration, especially when exposed to oxygen, temperature, and light. The nutritional quality of edible oils can be compromised by the instability and susceptibility to oxidative degradation. These problems affect the development of off-flavor, shelf stability, and sensory properties of products. Therefore, products containing lutein and edible oils should be protected from physical and chemical damage. The stability of lutein can be greatly improved with microencapsulation technology. Microencapsulation is a technique for encapsulating a solid or liquid active substance with a coating material to preserve chemical or physical properties which can be used in food systems. It can also be used to enhance the shelf life of various bioactive compounds of therapeutic importance. The aim of this study is to determine a suitable extraction method by which to obtain lutein from marigold flower using virgin coconut oil, to evaluate the cytotoxicity of marigold flower extract in retinal pigment epithelial cells, to optimize the encapsulation process for the development of microencapsulated marigold flower extract, and to develop a method for the analysis of lutein in microencapsulated marigold flower extract using ultrahigh-performance liquid chromatography-quadrupole-Orbitrap high-resolution mass spectrometry (UHPLC-Q-Orbitrap-HRMS). The use of virgin coconut oil, which was obtained via the cold-pressed method in this study, avoids the uses of organic solvents, alkali treatment, and heat processing as employed in the refining process of other vegetable oils’ production. Although the cold-pressed coconut oil is more expensive than the refined one, it is interesting to create a value-added product using virgin (cold-pressed) coconut oil to support a value-based economy. In this study, the developed microencapsulated marigold flower extract was evaluated for cytotoxicity in a human RPE cell strain, namely, ARPE-19 cells, which can provide information related to its safety *in vitro*. An MTT assay, which was one of the most versatile and standard assays, was performed to determine the cell viability.

## 2. Results and Discussion

### 2.1. Extraction Yield

The results showed that the yield of marigold flower extracts obtained using the ultrasonication method (COU) and microwave-assisted method (COM) were found to be 72.10 and 74.23%, respectively.

### 2.2. Lutein Content in Marigold Flower Extract

#### 2.2.1. Method Validation

- Specificity

**Figure 1:**
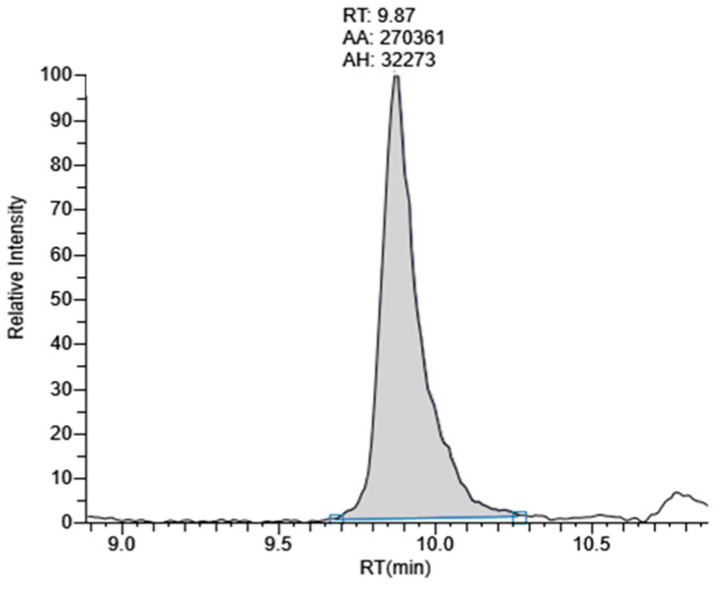
Chromatogram of standard lutein 500 ng/mL at the acquisition window for the SRM transition for lutein: *m*/*z* 568.4275→145.0927.

**Figure 2:**
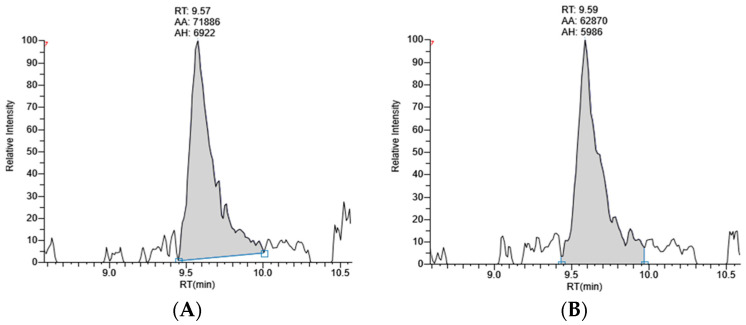
Chromatogram of lutein from marigold extract at 5 µg/mL obtained using coconut oil as an extraction solvent: (**A**) COM: marigold extracts with coconut oil via the microwave-assisted method; (**B**) COU: marigold extracts with coconut oil via the ultrasonic method. The acquisition window for the SRM transition for lutein: *m*/*z* 568.4275→145.0927.

A representative chromatogram of standard lutein at a concentration of 500 ng/mL, showing SRM transition at *m*/*z* 568.4275→145.0927, is shown in Figure 1. The retention time of lutein was 9.87 min. Chromatograms of the peak corresponding to lutein in marigold extracts at 5 µg/mL obtained using coconut oil as an extraction solvent and using microwave and ultrasonication extraction methods are shown in Figure 2. By comparing their MS/MS fragmentation with that of standard lutein, these peaks were identified as lutein.

- Linearity

**Figure 3:**
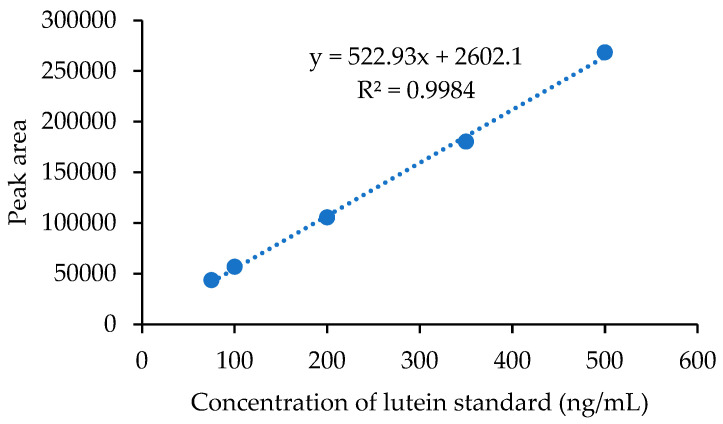
Standard curve of lutein.

The standard curve for the linearity between five concentrations of lutein and the corresponding peak areas was constructed. Linearity ranging from 75 to 500 ng/mL demonstrated good correlation (R^2^ = 0.9984) (Figure 3).

- Sensitivity

The limits of detection and quantification were 4.50 and 13.64 ng/mL, respectively. The obtained LOD and LOQ values via the LC–MS/MS method is considered suitable for the analysis of lutein.

- Accuracy

The acceptable ranges from ICH guideline [15] are 80 to 120% of the test concentration for the assay of a drug substance or a finished product. The result showed that the mean recovery of lutein was 90.53–125.20%, which was close to the acceptance criteria (Table 1).

- Precision

Intra-day and inter-day precision were evaluated. The RSDs of intra-day and inter-day precision of peak area were less than 7 and 5%, respectively (Table 2).

#### 2.2.2. Quantification of Lutein in Marigold Extract 

It was found that COM contained significantly higher lutein content than COU at 27.22 and 23.11 ng lutein/µg marigold extract, respectively (Table 3). Thus, COM was selected for the development of microencapsulated powder. This result is similar to the previous study, which compared the extraction methods including microwave, ultrasonic, and maceration methods. The results showed that the technique that gave the highest extraction efficiency was the microwave method, which took the shortest time. It is assumed that the microwave energy gave direct heating to the irradiation energy supplied to the solvent because the rotation of molecular dipoles and ionic conduction was induced by microwaves. The cells break and the substances can diffuse into the extraction solvent, whereas in ultrasonication, the cell wall is disrupted by cavitation, inducing the solvent to permeate the plant matrix [16]. Because of the heat generated in both the ultrasonic and microwave-assisted method, extraction time should be considered to prevent the degradation of lutein. The longer the heat exposure time, the higher possibility of lutein decomposition.

The sample preparation described in this study can be applied in the determination of lutein in the food matrix or finished products that contain vegetable oils as an ingredient, as well as in the biological testing to prove the health benefit of functional foods or dietary supplements in further studies. Moreover, the developed method for the determination of lutein in marigold extract using UHPLC-Q-Orbitrap-HRMS was successfully validated. This study expands the knowledge of the analytical method as applied to lutein in plants and raw materials of which a limited number of studies is currently available. 

### 2.3. Cytotoxicity of Lutein-Rich Fraction from Marigold Extracts in ARPE-19 Cell Line

**Figure 4:**
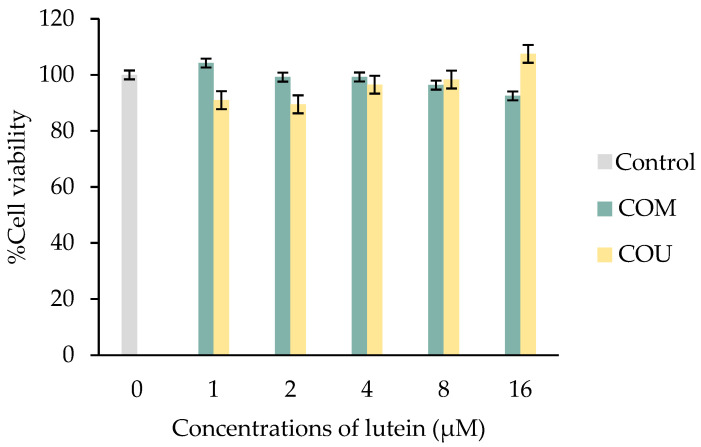
Cytotoxicity of lutein-rich fraction from marigold extracts in ARPE-19 cell line. COU: marigold extracts with coconut oil by ultrasonic method; COM: marigold extracts with coconut oil via the microwave-assisted method. Values were expressed as mean ± SD (*n* = 3).

The cytotoxic effects of the lutein-rich fraction from marigold extracts obtained via the microwave-assisted method and ultrasonic method are displayed in Figure 4. The percentage of cell viability treated with the lutein-rich fraction from marigold extracts were not significantly different from control (*p* < 0.05). It can be concluded that lutein-rich fractions from marigold extracts were non-toxic to the cells at the concentrations tested.

### 2.4. Microencapsulation of Marigold Extract

It was found that the emulsion of all formulations were stable. Phase separation was not detected. The result of powder characterization is shown in Table 4. The range of microencapsulation yield was 62.01 to 70.20%, which was not significantly different for all formulae. The moisture content of the microcapsules ranged from 3.83 to 4.88%, which is considered suitable for powdered food. This result agreed with San et al.’s 2022 study [17], which reported that when the wall-to-oil ratio increased, the moisture content also increased. However, there was no significant difference between F2, F3, and F4. A previous study reported that the wall-to-oil ratio and the type of wall material had a significant effect on moisture content. This may be due to the influence of the molecular size of the wall material, which may hinder the diffusion rate of particle shell formation. Therefore, the higher solid content may increase viscosity and slow the diffusion of water [17,18].

The percentages of microencapsulation efficiency (%ME) were in the range of 35.33 to 82.60% (Table 4). It was found that the %ME of four formulae were significantly different; F4, which had the highest wall-to-oil ratio, had the highest %ME of 82.60%. The lowest %ME was found in F1. The previous study indicated that %ME decreases as the wall-to-oil ratio decreases because the amount of wall material is insufficient to cover the water droplets that have formed. The droplets will have a chance to pool together, especially when the wall-to-oil ratio is low [17,19].

The lutein content in microencapsulated powder was analyzed by using UHPLC-Q-Orbitrap-HRMS and the result is shown in Table 4. F3 had the lowest lutein content when compared with F1, F2, and F4 at the concentration of 1 mg/mL.

**Figure 5:**
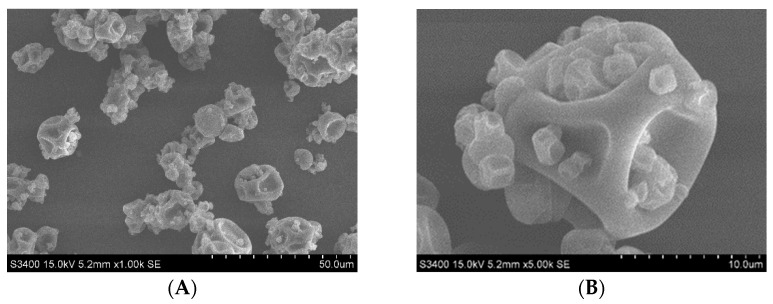
SEM images of microencapsulated powder of marigold extract prepared with MD/GA at 1:5 oil-to-wall ratios, 30% total solid content. Magnification at (**A**) 1000× and (**B**) 5000×.

The SEM analysis of the microencapsulated powder of the marigold extract revealed the appearance of F4 in Figure 5. The characteristics showed that the powder has dents without cracks on the surface. The difference in powder morphology was clearly demonstrated by comparing the microcapsule contraction. The contraction of the particle surface increased when the wall-to-oil ratio increased [17]. Previous studies have suggested that prolonged film formation and water evaporation during spray drying may affect surface dents [17,20]. In the overall powder morphology at 1000× (Figure 5), spray drying produced a wide range of particle sizes, which is in accordance with the study by Kurniawan et al., 2019 [21]; the lutein ester in coconut oil was encapsulated with maltodextrin and acacia gum at a ratio of 60:40; they suggested that the different particle sizes were associated with agglomeration. In addition, spray drying parameters may influence the formation of cavities in the particle surface [22]. Thereby, the suggested ratio of oil to wall from this study was 1:5; note that the optimum ratio of oil to wall according to the previous study was usually 1:2 to 1:3, depending on the purpose of the product and the processing conditions involved [23]. In general, lutein is readily degradable. Thus, further study on the stability of lutein in the extract and the microencapsulated powder is recommended.

## 3. Materials and Methods

### 3.1. Plant Materials

Marigold flowers *(T. erecta* L.) were purchased from Rai Ruen Rom Organic Farm (Chiang Rai, Thailand). The plant was identified by Dr. Sunisa Sangvirotjanapat, Project of Institute Establishment for Sireeruckhachati Nature Learning Park, Mahidol University, Nakhon Pathom, Thailand. Voucher specimen (No. PBM 005761) were deposited at the Department of Pharmaceutical Botany, Faculty of Pharmacy, Mahidol University. Marigold petals were separated from the flowers. The petals were washed and drained. Then, they were dried by hot air oven at 50 °C [24] until the moisture content was less than 10%. The dried petals were ground and stored in low humidity and protected from light until further use.

### 3.2. Chemicals and Reagents

Virgin coconut oil (Tropicana, Tropicana oil Co., Ltd., Nakhon Pathom, Thailand) was purchased from local supermarket in Bangkok. The standard lutein was purchased from ChemFaces, Wuhan, China. LC–MS/MS-grade methanol, acetonitrile, and formic acid were obtained from Fisher Scientific, Waltham, MA, USA. 

### 3.3. Determination of Suitable Extraction Method 

In previous studies, different vegetable oils were used as extraction solvents, including sunflower oil, soybean oil, and coconut oil. Marigold flower extracted via coconut oil obtained the highest content of lutein. Thus, coconut oil was selected to be used as the extraction solvent in this study. 

#### 3.3.1. Ultrasonic Extraction 

Dried marigold powder was suspended in coconut oil. The ratio of dried marigold powder and the oil was fixed at 1:5 (*w*/*w*), and the mixture was sonicated for 20 min in an ultrasonic bath (Emerson, St. Louis, MO, USA). Then, the oil part was collected and filtered via Whatman No.4. The marc was re-extracted twice. The extract was kept in a refrigerator until further used [25]. 

#### 3.3.2. Microwave-Assisted Extraction 

Dried marigold powder was suspended in coconut oil. The ratio of dried marigold powder and the oil was fixed at 1:5 (*w*/*w*), and the mixture was extracted in microwave oven for 1 min, operating at a maximum power of 900 W (Sanyo: EM-M200W, Tokyo, Japan). Then, the oil part was collected and filtered via Whatman No.4. The marc was re-extracted twice. The extract was kept in a refrigerator until further used [25].

### 3.4. Sample Preparation

The extracts obtained from the oil extraction need to be concentrated before further analysis. The oil was removed using a saponification method. The saponification method was performed by mixing 5 g of marigold extract with 10 mL of diethyl ether (Labscan, Dublin, Ireland). Then, 10 mL of ethanolic solution containing 10% KOH (Carlo Erba, Cornaredo, Italy) was added into the mixture and incubated at room temperature in the dark for 2 h. The saponifiable part was washed with water and the layer of diethyl ether was dried using a rotary evaporator. The dried sample of the lutein-rich fraction was kept at −20 °C in the dark until further used [21]. 

### 3.5. Determination of Lutein Content in Marigold Flower Extract 

LC–MS/MS analysis was carried out using Vanquish UHPLC. Mass detection was carried out using an Orbitrap Exploris 480 MS (Thermo Scientific, Bremen, Germany). The instruments were controlled by Xcalibur software version 4.4. The software was also used for data acquisition. The data processing was analyzed by TraceFinder software version 5.1. UHPLC chromatographic separations were performed on reversed-phase column Chromolith^®^ Performance RP-18e, 2.1 μm, 4.6 × 100 mm (Merck KGaA, Darmstadt, Germany). The HPLC condition using LC–MS/MS was as follows: mobile phase consisted of solvent A: 0.1% formic acid in water; and solvent B: the mixture of 0.1% formic acid in methanol and acetonitrile (ratio 1:1; Fisher Scientific, UK) [26]. The gradient elution was performed as follows: 0–0.5 min, 10% B; 3–15 min, 98% B; 15–25 min, 10% B. The chromatographic column and sample temperatures were set at 40 and 4 °C, respectively. Injection volume was 10 µL. The MS conditions were performed with electrospray ionization (ESI) in positive mode, operating in the SRM scan acquisition mode. The parameters for dissociation energy and collision energy were optimized to obtain the maximum relative amount of precursor ions and product ions as follows: ion transfer tube temp: 320 °C; vaporizer temp: 75 °C; sheath gas: 25 Arb; aux gas: 7 Arb; sweep gas: 0; RF lens: 50%; collision energy: 30%. The method validation was performed according to the ICH guideline [15] including specificity, sensitivity, linearity, accuracy, and precision. The calibration curves were plotted according to peak area versus concentration of lutein. The standard solution of lutein was dissolved in 0.1% formic acid in acetonitrile to obtain the concentrations of 75, 100, 200, 350, and 500 ng/mL. 

### 3.6. Cytotoxicity Study of Marigold Extract in ARPE-19 Cell Line

#### 3.6.1. Cell Culture

ARPE-19 (ATCC; CRL-2302, Manassas, VA, USA) cells were cultured in DMEM:F-12 (ATCC; cat.no. 30-2006, USA) medium containing 10% heat-inactivated FBS (Gibco; cat.no 10270-106, Waltham, MA, USA) and 1% penicillin–streptomycin (Sigma-Aldrich; cat.no P4333, St. Louis, MO, USA). They were incubated at 37 °C with 5% CO_2_. Cells were passaged when they reached 80% confluency. The range of cell passage numbers during these experiments was between 23 to 32 [27].

#### 3.6.2. Cell Viability Assay

Cell viability was determined via MTT assay. After treatment, the cells in a 96-well plate were washed with PBS (Merck; cat.no. 524650, Rahway, NJ, USA) twice and replaced with 100 µL culture medium. Then, 10 µL of 5 mg/mL MTT (Invitrogen; cat.no. M6494, Waltham, MA, USA) solution was added to each well and incubated at 37 °C with 5% CO_2_ for 4 h. After that, the MTT solution was removed. Twenty-five microliters of media and 50 µL of DMSO were added to each well, mixed properly, and the plate was incubated at 37 °C for 10 min. Finally, the absorbance of samples was read at 540 nm with a microplate reader.

#### 3.6.3. Cytotoxicity Assay

ARPE-19 cells were seeded in 96-well plates at a density of 2 × 10^4^ cells/well. Cells were treated with lutein-rich fractions from marigold extracts at concentrations equivalent to 1, 2, 4, 8, and 16 µM of lutein in a culture medium for 24 h. DMSO at the concentration of 1.36% in the media was used as control [28]. After the treatment, the MTT assay as described in 3.6.2. was carried out to determine cell viability. 

### 3.7. Development of Microencapsulated Marigold Flower Extract

The formulae for microencapsulation are shown in Table 5, fixing the total solid content at 30%. The wall material was dissolved in deionized water at room temperature. Then, marigold flower extract was added, and the mixture was homogenized at 11,000 rpm for 8 min. The encapsulated product was kept at room temperature in the dark prior to spray drying. The spray drying process was carried out using a mini spray dryer (Buchi: B-290, Flawil, Switzerland) to obtain the powder form of microencapsulated marigold flower extract at the following parameters: 170 °C of the inlet temperature, 100% aspiration rate, 10% pump, and feed rate at 3.33 mL/min [20]. 

Emulsion characterization and powder characterization of microencapsulated marigold flower extract were studied as mentioned below. 

#### 3.7.1. Emulsion Characterization 

The stability of emulsions was analyzed via visual observation. Phase separation was observed at 24 h after emulsion preparation, i.e., placing 100 mL of the emulsions before spray drying them in a cylinder and storing them at room temperature. Emulsion stability was determined using equation below [29]:

$$\%\;\mathrm{Creaming}\;\mathrm{index}=\frac{\mathrm{Upper}\;\mathrm{phase}\;\mathrm{height}\;}{\mathrm{Initial}\;\mathrm{height}\;\mathrm{of}\;\mathrm{emulsion}}\;\times 100$$

#### 3.7.2. Powder Characterization

- Moisture Content

Powder (0.5 g) was spread on the sample pan of the infrared moisture analyzer (Radwag: MA 50.R, Radom, Poland). The chamber was heated to 105 °C, and drying was stopped when the sample achieved constant weight [30].

- Microencapsulation Yield

The microencapsulation yields of the microcapsules were the mass ratio of the total solids in the microcapsule to the initial solids in emulsion [31]:

$$\mathrm{Microencapsulation}\;\mathrm{yields}\;(\%)=\frac{\mathrm{The}\;\mathrm{total}\;\mathrm{solid}\;\mathrm{in}\;\mathrm{microcapsule}\;}{\mathrm{The}\;\mathrm{initial}\;\mathrm{solid}\;\mathrm{in}\;\mathrm{emulsion}}\;\times 100$$

- Microencapsulation Efficiency
  o Surface Oil

Two grams of powder were mixed with 5 mL hexane in a centrifuge tube for 2 min. The mixture was filtered through a filter paper, and the solid particles were washed twice with 20 mL of hexane. Then, hexane was removed under the fume hood. Surface oil was collected from the mass difference [17].

o
Total Oil

One gram of powder was mixed with 5 mL of deionized water for 2 min, and 25 mL of hexane/propan-2-ol (3:1 *v*/*v*) was added. The mixture was mixed via a vortex mixer for 5 min and centrifuged at 3000 rpm for 30 min. The organic phase (upper layer) was collected. The aqueous phase was re-extracted twice with the same volume of extractant. All organic phases were mixed with sodium sulfate anhydrous and filtered through Whatman No.1. Then, the organic solvent was removed under the fume hood. The total oil was collected from the mass difference [17].

$$\mathrm{Microencapsulation}\;\mathrm{efficiency}\;(\%)=\frac{\mathrm{Total}\;\mathrm{oil}\;-\;\mathrm{Surface}\;\mathrm{oil}\;}{\mathrm{Total}\;\mathrm{oil}}\;\times 100$$

- Scanning Electron Microscopy (SEM)

SEM (Hitachi; S34000, Tokyo, Japan) was used to study the morphology of microencapsulated powder by sprinkling the microencapsulated powder onto a double-sided conductive tape attached to aluminum stub. Then, excess powder was removed with a blower. The microencapsulated powder was coated with gold on the surface using an ion coaster under vacuum. The SEM analysis was performed at 15 kV, and magnifications were measured at 1000× and 5000× [17].

#### 3.7.3. Quantification of Lutein in Microencapsulated Marigold Flower Extract

The content of lutein in the microencapsulated powder was determined using UHPLC-Q-Orbitrap-HRMS following the methods described in Section 3.4. and Section 3.5.

### 3.8. Statistical Analysis

In this study, each experiment was performed at least three times. Data were shown as mean ± SD and analyzed via one-way ANOVA. The statistical significance was set at *p*-values less than 0.05.

## 4. Conclusions

The extraction of lutein from marigold flowers using coconut oil as an extraction solvent via the microwave-assisted method can be used as an alternative, green method because this method is less time-consuming and environmentally friendly. The LC–MS/MS method for the determination of lutein in marigold extract was successfully developed and validated. These results add to the limited number of studies on analytical methods of lutein in plant and raw material. Therefore, the developed LC–MS/MS method can be applied for the determination of lutein and the quality control of the product containing lutein in the future. The marigold flower extract at the concentration equivalent to 16 µM of lutein was found to be non-toxic to ARPE-19 cells. This result suggests the *in vitro* safety of marigold flower extract; however, further studies in animal models on toxicity and efficacy related to eye health are needed. For the development of the microencapsulated marigold extract, the ratio of oil to wall at 1:5 had the highest encapsulation efficiency and the highest lutein content. The encapsulation technique could preserve the bioactive compound, lutein, in the finished product. This study revealed the potential of the developed microencapsulated marigold extract as a promising functional ingredient. This is considered a value-added product from marigold flower and coconut oil.

## Figures and Tables

**Table 1:** Recovery tests for lutein in marigold extract.

Compound	Standard Addition (%)	Recovery Rate (%)
Lutein	50	90.53 ± 2.00
	100	101.96 ± 169
	150	125.20 ± 2.18

**Table 2:** Precision tests for lutein from standard lutein.

Conc. Of Lutein (ng/mL)	RSD (%)	
	Intra-Day	Inter-Day
75	1.78	3.15
200	0.52	1.64
500	6.46	4.62

**Table 3:** Lutein content in marigold extracts.

Sample	Compound	Amount (ng Lutein/µg Marigold Powder)	%RSD	Rt (min)	%RSD
COM	Lutein	27.22 ± 1.17 ^a^	4.31	9.58 ± 0.01	0.12
COU	Lutein	23.11 ± 0.49 ^b^	2.11	9.60 ± 0.01	0.10

^a,b^: Values were expressed as mean ± SD (*n* = 3). The values followed by different letters in the same column are significantly different (*p* < 0.05).

**Table 4:** Powder characterization of microencapsulated powder from marigold extracts.

Formula	Oil-to-Wall	Moisture Content (%)	Yield (%)	ME (%)	Lutein Content (µg Lutein/g Powder)
F1	1:2	3.83 ± 0.02 ^b^	62.01 ± 4.35 ^a^	35.33 ± 3.21 ^d^	6.11 ± 0.11 ^a^
F2	1:3	4.67 ± 0.10 ^a^	67.52 ± 5.75 ^a^	52.16 ± 4.39 ^c^	5.98 ± 0.11 ^a^
F3	1:4	4.86 ± 0.09 ^a^	65.71 ± 5.75 ^a^	72.29 ± 0.96 ^b^	4.69 ± 0.25 ^b^
F4	1:5	4.88 ± 0.23 ^a^	70.20 ± 6.22 ^a^	82.60 ± 1.49 ^a^	6.18 ± 0.17 ^a^

ME, microencapsulation efficiency. Values were expressed as mean ± SD (*n* = 3). The values followed by different letters in the same column are significantly different (*p* < 0.05).

**Table 5:** Formulations for microencapsulated marigold flower extract.

Formula	Oil-to-Wall	Ingredients (%*w*/*w*)			
		Marigold Flower Extract	Maltodextrin	Gum Acacia	Deionized Water
F1	1:2	10.00	6.00	14.00	70
F2	1:3	7.50	6.75	15.75	70
F3	1:4	6.00	7.20	16.80	70
F4	1:5	5.00	7.50	17.50	70

## Data Availability

Data are contained within the article.
